# Differences in the Ovine *HSP90AA1* Gene Expression Rates Caused by Two Linked Polymorphisms at Its Promoter Affect Rams Sperm DNA Fragmentation under Environmental Heat Stress Conditions

**DOI:** 10.1371/journal.pone.0116360

**Published:** 2015-02-11

**Authors:** Judit Salces-Ortiz, Manuel Ramón, Carmen González, M. Dolores Pérez-Guzmán, J. Julián Garde, Olga García-Álvarez, Alejandro Maroto-Morales, Jorge H. Calvo, M. Magdalena Serrano

**Affiliations:** 1 INIA, Carretera de La Coruña Km. 7.5, 28040, Madrid, Spain; 2 CERSYRA, Av. Del Vino 10, 13300, Valdepeñas, Spain; 3 SaBio IREC (CSIC–UCLM–JCCM) Campus Universitario S.N., 02071, Albacete, Spain; 4 Unidad de Tecnología en Producción Animal, CITA, 59059, Zaragoza, Spain; University of South Florida College of Medicine, UNITED STATES

## Abstract

Heat shock (HS) is one of the best-studied exogenous cellular stresses. Almost all tissues, cell types, metabolic pathways and biochemical reactions are affected in greater or lesser extent by HS. However, there are some especially thermo sensible cellular types such as the mammalian male germ cells. The present study examined the role of three INDELs in conjunction with the -660G/C polymorphism located at the *HSP90AA1* promoter region over the gene expression rate under HS. Specially, the -668insC INDEL, which is very close to the -660G/C transversion, is a good candidate to be implied in the transcriptional regulation of the gene by itself or in a cooperative way with this SNP. Animals carrying the genotype II-668 showed higher transcription rates than those with ID_-668_ (FC = 3.07) and DD_-668_ (FC = 3.40) genotypes for samples collected under HS. A linkage between gene expression and sperm DNA fragmentation was also found. When HS conditions were present along or in some stages of the spermatogenesis, alternative genotypes of the -668insC and -660G/C mutations are involved in the effect of HS over sperm DNA fragmentation. Thus, unfavorable genotypes in terms of gene expression induction (ID_-668_GC_-660_ and DD_-668_GG_-660_) do not produce enough mRNA (stored as messenger ribonucleoprotein particles) and Hsp90α protein to cope with future thermal stress which might occur in posterior stages when transcriptional activity is reduced and cell types and molecular processes are more sensible to heat (spermatocytes in pachytene and spermatids protamination). This would result in the impairment of DNA packaging and the consequent commitment of the events occurring shortly after fertilization and during embryonic development. In the short-term, the assessment of the relationship between sperm DNA fragmentation sensitivity and ram’s fertility will be of interest to a better understanding of the mechanisms of response to HS and its consequences on animal production and reproduction performance.

## Introduction

Heat shock (HS, also known as heat stress or hyperthermia) is one of the best-studied exogenous cellular stresses. The cellular response to HS utilizes ancient molecular networks that are based primarily on the action of stress-induced heat shock proteins (HSP) and heat shock factors. All eukaryotes produce at least one heat inducible protein with molecular sizes that ranges from 80 to 90 kDa [1]. Members of the *HSP90* gene family have undergone major duplication events, which led to two known cytoplasmic isoforms, namely Hsp90α (also named *Hsp90AA1*) or inducible form and Hsp90β (also named *Hsp90AB1*) which is the constitutive one. Whereas Hsp90β is more or less constitutively and ubiquitously expressed, the expression of Hsp90α is heat-inducible and more tissue specific [2]. Hsp90α predominates in the brain and testis and is necessary for the spermatogenesis and meiotic progression in testis [3]. The transcriptional enhancement of Hsp90α is mainly due to the HSF1 (heat shock factor 1), but many other heat stress related transcription factors also regulate gene expression in response to environmental stress [1, 4].

Almost all tissues, cell types, metabolic pathways and biochemical reactions are affected in greater or lesser extent by heat stress. However, there are some cellular types specially thermo sensible such as the mammalian male germ cells. The sensitivity of these cells to environmental heat has been extensively studied [5–7]. A reduction in sperm DNA integrity has been described in human [8], mice [9, 10] and rams [11] as well as alterations in DNA, RNA and protein synthesis, and abnormal chromatin packing in mice [9, 10, 12] under heat stress conditions. Proper compaction and structure of DNA has been reported to have important functional roles, being essential for DNA replication and embryonic development [13, 14]. In a recent work of our group [15] it has been observed that sperm DNA fragmentation in rams increases over a threshold of 30°C of maximum temperature (temperature-humidity index THI > 22) at some stages of the spermatogenesis process in animals carrying the GG_-660_ genotype of the -660G/C SNP (rs397514116). As GG_-660_ genotype has been associated to lower levels of *HSP90AA1* expression under heat stress conditions [16] it has been suggested that suboptimal amounts of *HSP90AA1* mRNA make spermatozoa DNA of these animals more susceptible to be fragmented.

In sheep, the gene encoding the *HSP90AA1* isoform has been extensively studied [17–19]. At the promoter of this gene 14 polymorphisms (11 SNPs and 3 INDELs) have been described [17, 19]. One of these mutations, -660G/C SNP (rs397514116), have been related with the *scrapie* incubation period [18]; the adaptation of this species to differential thermal conditions [20]; and as noted above, ram’s sperm DNA fragmentation levels [15]. All these facts, which have been observed under thermoneutral, heat stress and missfolding stress conditions, seem to be consequence of the differences in the gene expression profile caused by this SNP located at the gene promoter [16, 20]. Therefore, it is reasonable to hypothesize that these polymorphisms are affecting some mechanisms of the gene transcription regulation (transcription factor binding affinity, structural features, etc) leading to differences in the RNA synthesis efficiency. However, as -660G/C is in linkage disequilibrium with other close mutations [16] it is not possible to determine which is/are the causal mutation/s responsible/s of the expression differences observed. In addition, until today, the possible relation with the expression rate of the gene of the three INDELs at positions -667insC, -668insC and -516insG (rs397514115.1, rs397514115.2 and rs397514268, respectively) located at the *HSP90AA1* gene promoter have been never studied.

The present study was designed to better understanding the role of the *HSP90AA1* gene in the response of animals to heat stress. Specifically, we aimed to know more about the expression patterns of the gene and the consequences in sperm DNA fragmentation levels derived from an exposure to a heat stress environment and depending on the genotype of males for the -660G/C SNP and the three INDELs located at the *HSP90AA1* gene promoter. For that, this work included two specific objectives: 1) study the effect of some INDELs located at the *HSP90AA1* gene promoter over its expression rate under heat stress and control conditions by q-PCR; 2) Test the effect of the INDELs along with the -660G/C SNP over ram’s sperm DNA fragmentation under different environmental conditions.

## Material and Methods

### Ethics Statement

The current study has been approved by the INIA Scientific Ethic Committee (IACUC). Animal manipulations were performed according to the Spanish Policy for Animal Protection RD 53/2013, which meets the European Union Directive 63/2010/EU about the protection of animals used in experimentation.

### Weather data

As a way to examine the role of -660G/C and -668insC polymorphisms on the observed effect of heat on sperm chromatin integrity [15, 16], climate and sperm DNA status data were gathered from March to October.

Meteorological data was provided by the Irrigation Advisory Service for Farmers (SIAR) in Castilla-La Mancha. Table 1 shows climate parameters at day of blood and sperm samples collection, and also the average from 2 days before to the day of samples collection. The meteorological data set consisted of hourly measures of temperature (°C) and relative humidity (%) on 245 days from March to October 2010. Daily average temperature (Tave, °C), daily maximum temperature (Tmax, °C) and daily average relative humidity (RH, %) were calculated from these hourly records. A temperature-humidity index (THI) was also calculated as proposed by Marai et al. [21] by combining daily average temperature (Tave) in °C with daily average relative humidity (RH) %∙0.01:

THI=Tave-[(0.31-0.31⋅RH)⋅(Tave-14.4)]

**Table 1:** Climate parameters in days of blood and sperm samples collection.

**blood collection date**	**Day**	**AvT**	**MaT**	**MiT**	**Rh**	**Rhmax**	**Rhmin**	**THIavr**	**THImax**	**treatment ID**
23/03/2010	0	11.6	19.9	3.8	69.0	92.6	37.2	11.87	19.77	Control
	2 to 0	11.9	17.8	6.0	74.8	93.0	46.4	12.09	17.54	
05/07/2010	0	26.8	35.0	16.8	39.4	63.9	19.8	24.47	32.69	July
	2 to 0	25.5	33.1	17.4	44.9	74.1	22.3	23.62	29.88	
03/08/2010	0	24.7	34.4	16.6	49.4	89.3	21.0	23.08	33.74	August 1
	2 to 0	26.1	35.1	16.4	41.3	77.0	17.2	23.95	31.34	
09/08/2010	0	27.3	33.8	22.2	50.0	71.5	28.2	25.30	32.09	August 2
	2 to 0	27.4	35.4	20.3	48.6	76.5	23.23	25.36	32.08	
sperm collection date										
01/03/2010	0	7.8	11.9	4.8	79.5	90.3	58.5	8.22	11.98	
	2 to 0	10.4	16.0	6.4	75.5	91.4	48.0	10.6	15.8	
19/05/2010	0	18.4	26.6	8.1	46.1	82.8	24.1	17.73	25.95	
	2 to 0	16.6	25.7	6.0	50.4	87.2	22.4	16.2	24.0	
29/06/2010	0	23.7	32.8	16.9	58.7	91.6	24.4	22.51	32.32	
	2 to 0	23.7	32.9	15.4	49.7	81.0	19.9	22.2	29.9	
14/07/2010	0	24.9	32.6	17.7	42.2	67.6	20.1	23.02	30.77	
	2 to 0	26.5	34.5	17.9	33.7	58	15.1	24.0	30.3	
27/07/2010	0	26.2	36.4	15.4	45.6	87.8	13.8	24.21	35.57	
	2 to 0	25.6	35.3	16.3	38.8	71.3	15.1	23.5	31.3	
10/08/2010	0	27.5	36.6	19.1	48.6	77.0	24.3	25.41	35.02	
	2 to 0	27.4	35.5	20.3	48.5	75.1	25.3	25.3	32.1	
24/08/2010	0	23.0	31.5	15.9	52.2	86.4	23.6	21.73	30.78	
	2 to 0	24.7	33.3	16.6	48.7	82.6	19.3	23.1	30.4	
05/10/2010	0	14.4	22.7	5.6	67.0	96.3	31.8	14.40	22.60	
	2 to 0	15.1	21.0	8.5	66.4	94.6	39.0	15.0	20.3	

Data are the climate values the day of collection. For blood collection dates, the mean from 2 days before to the day of collection is also presented.

Data from: Manzanares (Ciudad Real) Meteorological Station, coordinates 654m–38° 59’47N–03° 22’23W (http://crea.uclm.es/siar). AvT = average temperature (°C); MaT = maximum temperature (°C); MiT = minimum temperature (°C); Rh = average relative humidity (%); Rhmax = maximum relative humidity (%); Rhmin = minimum relative humidity (%); THIavr = THI calculated with the average temperature and relative humidity; THImax = THI calculated with the maximum temperature and the maximum relative humidity; Temperature humidity index (THI) calculated as THI = T-[0.31(1-RH)(T-14.4)], T = temperature in °C; RH = relative humidity/100 [21].

### Animal material, nucleic acid isolation, DNA amplification and INDELs genotyping.

Genomic DNA from 120 rams pertaining to the experiment carried out by Salces-Ortiz and coworkers in 2013 [16] were used to genotype the INDELs located at the *HSP90AA1* promoter. The polymerase chain reaction was performed from 100ng of genomic DNA using CERTAMP complex amplifications kit chemistry (Biotools, Madrid, Spain) with specific primers (S1 Table). The resulting PCR fragment was purified with ExoSAP-IT (USB Corporation) and sequenced with specific primers (S1 Table). S1 Fig. shows the *HSP90AA1* promoter sequence (DQ983231.1 Ovis aries heat shock protein alpha (HSPCA) gene) in which polymorphisms (SNPs and INDELs) are identified as well as some regulatory motifs, i.e. heat shock element (HSE) and TATA box.

### Linkage disequilibrium analysis

PLINK software [22] (http://pngu.mgh.harvard.edu/purcell/plink/) was used to estimate linkage disequilibrium among the three INDELs here detected and the remaining polymorphisms previously described [17] using r^2^, the squared correlation based on genotypic allele counts [23].

### Detection of putative transcription factor (TF) binding sites in ovine HSP90AA1 promoter

Putative TF binding sites were *in silico* predicted using Chip Mapper (Multi-genome Analysis of Positions and Patterns of Elements of regulation) [24].

### q-PCR reactions and expression analyses

Animals, biological samples, environmental conditions, experimental designs, q-PCR reactions and statistical methods of analysis were the same that those previously described in [16]. Briefly, the experiment consisted on the analysis of *HSP90AA1* RNA content in blood samples collected from 120 rams in four time points with different climatic conditions. q-PCR assays were performed per triplicate and using the *HSP90AB1* gene as housekeeping to normalize *HSP90AA1* expression results. Statistic methodology to analyze differences in the expression rate of alternative genotypes of some polymorphisms located at the gene promoter was that described by Steibel et al. [25].

### Semen samples collection and Sperm DNA fragmentation assay

Animals (61 rams selected from the 120 used in the expression analyses), biological samples, environmental conditions and sperm DNA fragmentation assay (SCSA) data were those used in the work of Ramón and colleagues [15]. Briefly, a total of 7 collections per male were carried out, from March to October as a way to ensure that sperm were exposed to different environmental conditions of temperature and humidity. After collection, sperm samples were incubated at 37°C during 48 hours, and the sperm DNA fragmentation was assessed after collection (0 h) and after 24 and 48 h. Sperm incubation at 37°C has the aim to mimic the environmental circumstances existing at ewe reproductive track. The SCSA assay expressed the extent of DNA denaturation in terms of DNA Fragmentation Index (DFI), which is the ratio of red to total (red plus green) fluorescence intensity, i.e. the level of denatured DNA over the total DNA [26]. The DFI value was calculated for each sperm cell in a sample, and the resulting DFI frequency profile was obtained. Based on the results of the study referred above, only total DNA fragmentation index (tDFI) values were used as dependent variable related to the extent of sperm DNA fragmentation. The tDFI was defined as the percentage of spermatozoa with a DFI value over 25%. Table 2 shows average tDFI values in each incubation stage for the non-heat stress (March to May) and heat stress (June to October) semen collections.

**Table 2:** Sperm DNA fragmentation levels (tDFI) at three incubation times for the non-heat stress (NHS) and heat stress (HS) semen collection periods.

**Semen collection period**	**0 h**	**24 h**	**48 h**
NHS	4.36 ± 0.12	4.64 ± 0.15^a^	6.67 ± 0.77^a^
HS	5.03 ± 0.31	6.75 ± 0.38^b^	12.27 ± 0.81^b^

Data are mean ± standard errors.

tDFI = total DNA fragmentation index (percentage of spermatozoa with a DNA Fragmentation Index (DFI) value over 25%.)

NHS = non-heat stress period (semen collections from March to May)

HS = heat stress period (semen collections from June to October)

^a–b^: different superscript letters within column indicate significant differences between semen collection periods [21].

### Association analyses among HSP90AA1 genotypes and sperm DNA fragmentation

To assess the effect of heat load on sperm DNA integrity of animals carrying alternative genotypes for the polymorphisms studied, we have examined the relationship among tDFI values and average THI, as suggested in Ramon et al. [15]. Thus, in the present study we compare the degree of sperm DNA fragmentation between genotypes in response to weather conditions from the day 60 previous to semen collection to the day of collection. The aim was to identify those stages of the spermatogenesis in which certain genotypes are more susceptible to heat stress and to identify which polymorphisms play a more important role. For this, a linear mixed-effects model including genotype, sperm incubation time (0h, 24h and 48h), the THI (Temperature Humidity Index) and their interactions as fixed effects and male as random effect were conducted. The model was as follow:

$$y_{ijkl}=\mu +IT_{j}+f{\left(T\right)}_{k}+G\times f{\left(T\right)}_{lk}+a_{i}+e_{ijkl}$$

where:

*y_ijkl_*: tDFI measure
*µ*: global mean
*IT_j_*: sperm incubation time (3 levels: 0, 24 and 48 h)
f(T)_k_: effect of THI for each of the 60 days prior sperm collection
*G×*f*(T)_lk_*: genotype-temperature effect interaction
*a_i_*: male (61 levels)
*e_ijkl_*: heterogeneous random residual error ~ N(0, óei2)

Since it was expected that the effect of temperature on tDFI values was revealed from a threshold, the following function was used to model the temperature effect:

$$f{\left(T\right)}_{k}=\left\{\begin{array}{l} 0\text{, if }THI\leq k\\ b(THI-k)\text{, }otherwise \end{array}\right\}$$

where, *k* is the selected threshold. A threshold value of 22 was used, based on the THI heat stress categories reported by Marai et al. [21] and the results previously obtained for this breed [15]. As genotype (*G*_l_), two polymorphisms located at the promoter of the *HSP90AA1* gene were considered and three independent mixed models analyses were conducted based on the genotype included in the model: (i) the SNP G/C-660; (ii) the INDEL-668insC; and (iii) their interaction. Heterogeneous residual variance for the effect of sperm incubation time was also considered. Multiple comparisons among genotypes were conducted for each day, from the day 60 prior to semen collection to date of collection (N = 61), and two scenarios were considered: a Non Heat Stress (NHS) (sperm collections from March and May 2010) and Heat Stress (HS) (sperm collections from June, July, August and October) conditions.

Statistical analysis was performed using the R 3.0.3 statistical language [27]. For multiple comparisons analyses, Bonferroni correction was considered.

## Results

### Genotype and allele frequencies

Table 3 shows genotypes (a) and allele (b) frequencies of the three INDELs, (-667insC, -668insC and -516insG), and the -660G/C SNP located at the *HSP90AA1* promoter in the 120 rams of Manchega breed used in the expression studies [16] and also for the subset of 61 rams (from these 120) used in the sperm DNA fragmentation assay [15]. For the INDEL -667insC very low frequency of the I_-667_ allele was found. There was only one animal with the ID_-667_ genotype (being I insertion and D deletion) among the 120 rams of the expression analyses. All animals included in the sperm DNA fragmentation assay were homozygous DD_-667_. Therefore, this polymorphism was removed in subsequent analyses. However, the frequency of this polymorphism in the Manchega breed is somewhat greater than that here observed, 0.972 for the D_-667_ allele and 0.027 for the I_-667_ one (data not shown from 48 parent-offspring trios). Also, the INDEL -516insG showed quite extreme frequencies, being the D_-516_ allele the most frequent, 82%, in the samples studied, and was also not included either in the DNA fragmentation assay.

**Table 3:** Genotype and allele frequencies of the three INDELs, -668insC rs397514115.2, -667insC rs397514115.1 and -516insG rs397514268, and the SNP -660G/C rs397514116 located at the *HSP90AA1* promoter in the 120 rams of Manchega breed used in the expression studies and in the 61 rams of them used in sperm DNA fragmentation assays (I = insertion; D = deletion) [15].

**Marker**	**rs#^1^**	**postion at DQ983231.1 Genbank sequence**	**Genotype**	**rams expression (N = 120)**	**rams expression freq**	**rams fragmentation (N = 61)**	**rams fragmentation freq**
-668insC	rs397514115.2	g.667_668insC	II	5	0.042	2	0.033
			ID	23	0.192	13	0.213
			DD	92	0.767	46	0.754
-667insC	rs397514115.1	g.666_667insC	II	0	0	0	0
			ID	1	0.008	0	0
			DD	119	0.992	61	1
-660G/C	rs397514116	g.660 G>C	GG	41	0.342	21	0.344
			CG	39	0.325	20	0.328
			CC	40	0.333	20	0.328
-516insG	rs397514268	g.515_516insG	II	2	0.017	1	0.016
			ID	39	0.325	21	0.344
			DD	79	0.658	39	0.639
**Marker**	**rs#^1^**	**postion at DQ983231.1 Genbank sequence**	**Genotype**	**rams expression (2N = 240)**	**rams expression freq**	**rams fragmentation (2N = 122)**	**rams fragmentation freq**
-668insC	rs397514115.2	g.667_668insC	I	33	0.138	17	0.139
			D	207	0.863	105	0.861
-667insC	rs397514115.1	g.666_667insC	I	1	0.004	0	0
			D	239	0.996	122	1
-660G/C	rs397514116	g.660 G>C	G	121	0.504	62	0.508
			C	119	0.496	60	0.492
-516insG	rs397514268	g.515_516insG	I	43	0.179	23	0.189
			D	197	0.821	99	0.811

^1^ rs#= reference SNP ID number

### Linkage disequilibrium estimation among 11 polymorphisms of the HSP90AA1 promoter

Table 4 shows the matrix of r^2^ values among the 11 polymorphisms (see S1 Fig., for polymorphisms nomenclature) detected at the *HSP90AA1* promoter. Moderate LD values (from 31% to 33%) were found between pairs -516insG_-528A/G, -516insG_-660G/C and -516insG_-704insAA. The -667insC showed a 40% of LD with the SNP -444A/G and the -668insC showed LD values from 19% to 26% with -528A/G, -660G/C and the -704insAA. Among the three INDELs very low r^2^ values were found.

**Table 4:** Linkage disequilibrium values (r2) among the 11 polymorphisms detected at the *HSP90AA1* promoter.

	**-444A/G**	**-468G/T**	**-516insG**	**-522A/G**	**-524G/T**	**-528A/G**	**-601A/C**	**-660G/C**	**-667insC**	**-668insC**	**-704insAA**
-444A/G	1										
-468G/T	0.006	1									
-516insG	0	0.003	1								
-522A/G	0.005	0.003	0.019	1							
-524G/T	0.005	0.901	0.009	0.007	1						
-528A/G	0.045	0.027	0.333	0.011	0.018	1					
-601A/C	0.005	0.619	0.006	0.007	0.689	0.021	1				
-660G/C	0	0.032	0.314	0.038	0.023	0.584	0.025	1			
-667insC	0.398	0.001	0.004	0.001	0.001	0.012	0.001	0.013	1		
-668insC	0	0.003	0.001	0.001	0.002	0.199	0.001	0.251	0.016	1	
-704insAA	0	0.035	0.335	0.041	0.025	0.601	0.027	0.941	0.013	0.264	1

### Analysis of gene expression for isolate and combined genotypes of INDELs and -660G/C

In the present study, we have examined gene expression rates for alternative genotypes of the INDELs -668insC and -516insG conjunction with the -660G/C polymorphism, since in a previous work [16] animals carrying alternative genotypes of -660G/C temperature dependent differences in *HSP90AA1* gene expression rate were showed. The following contrasts between genotypes within treatments (Control, July, August 1 and August 2) where considered: **a)** contrasts between -668insC genotypes (DD_-668_, ID_-668_ and II_-668_; being I insertion and D, deletion); **b)** contrast between -516insG (DD_-516_, ID_-516_ and II_-516_ genotypes); **c)** contrasts between the existent combined genotypes -668insC_-660G/C (DD_-668_CC_-660_; DD_-668_CG_-660_; DD_-668_GG_-660_; ID_-668_CC_-660_; ID_-668_CG_-660_; II_-668_CC_-660_) **d)** contrasts between the existent combined genotypes -660G/C_-516insG (CC_-660_DD_-516_; CC_-660_ID_-516_; CC_-660_II_-516_; CG_-660_DD_-516_; CG_-660_ID_-516_; GG_-660_DD_-516_) and **e)** contrasts between the existent combined genotypes -668insC_-516insG (DD_-668_DD_-516_; ID_-668_DD_-516_; II_-668_DD_-516_; DD_-668_ID_-516_; ID_-668_ID_-516_; DD_-668_II_-516_).

**Figure 1:**
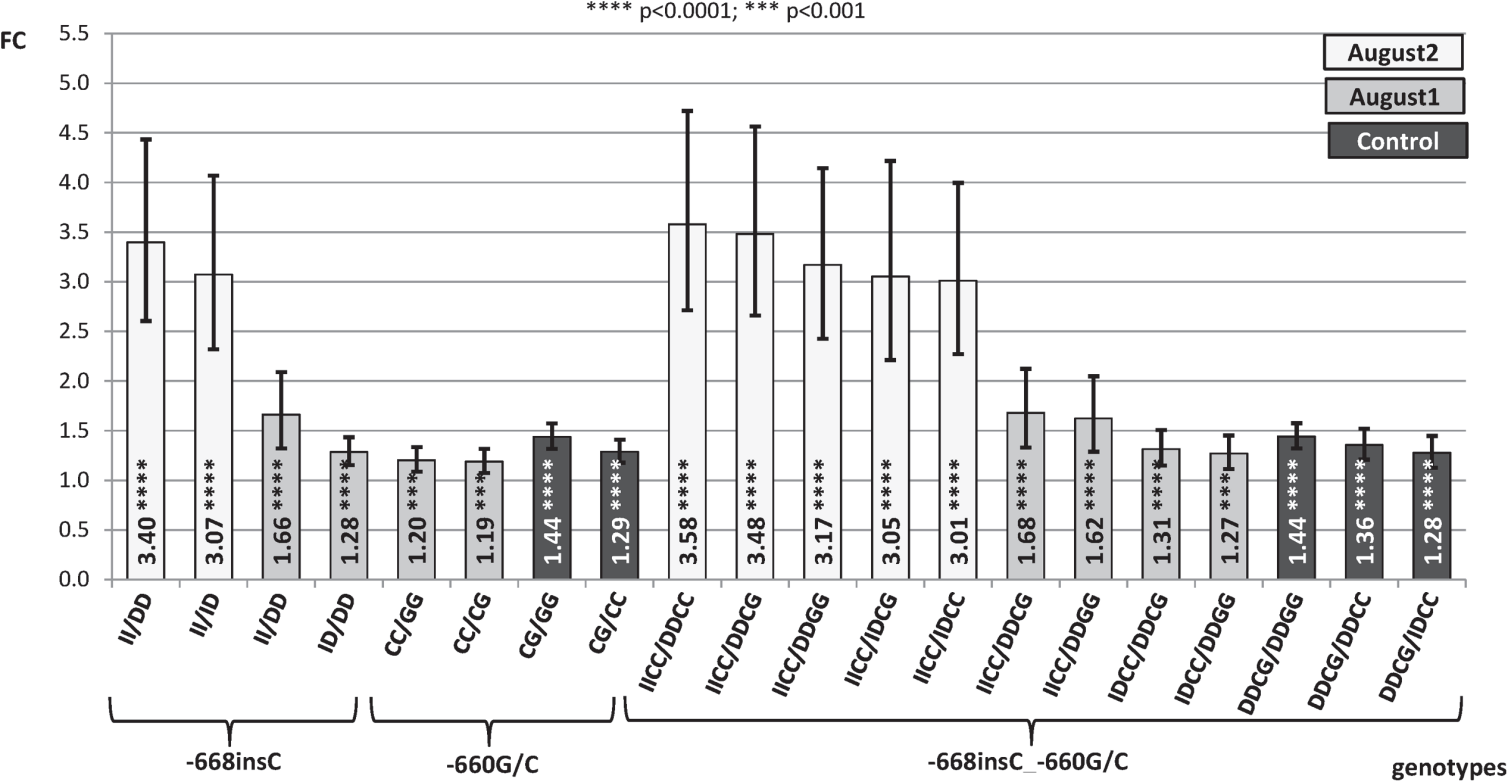
Fold change (FC) for the contrast among alternative genotypes of -668insC, -660G/C and combined genotypes -668insC_-660G/C of the *HSP90AA1* promoter within treatment normalized by *HSP90AB1*. Segments indicate the 95% confidence interval (FC_up_-FC_low_). In abscissa the FC, in ordinate genotype contrasts (I = insertion; D = deletion).

**a) Contrasts between alternative genotypes of the -668insC.** Fig. 1 shows the results for contrasts comparing cytosine insertion genotypes. Only high statistically significant contrasts were showed. We could observe differences in expression between genotypes only for samples collected in August 1 and August 2, when maximum environmental temperatures exceeded 33°C (Table 1). The homozygote genotype for the insertion (II_-668_) showed much higher expression rates (p<0.0001) than the heterozygote ID_-668_ (FC = 3.07) and the homozygote DD_-668_ (FC = 3.40) for samples collected in August 2 (average temperature = 27.3°C, maximum temperature = 33.8°C and minimum temperature = 22.2°C). For samples collected in August 1 (average temperature = 24.7°C, maximum temperature = 34.4°C and minimum temperature = 16.6°C) lower differences among genotypes than in the previous case were found. Thus, animals carrying the II-_668_ genotype showed higher expression rate (p<0.0001) than those with DD_-668_ (FC = 1.66). In this case also the heterozygote (ID_-668_) had significant (p<0.0001) higher expression levels than the DD_-668_ one (FC = 1.28) but no differences were observed between II_-668_ and ID_-668_ genotypes.

**Figure 2:**
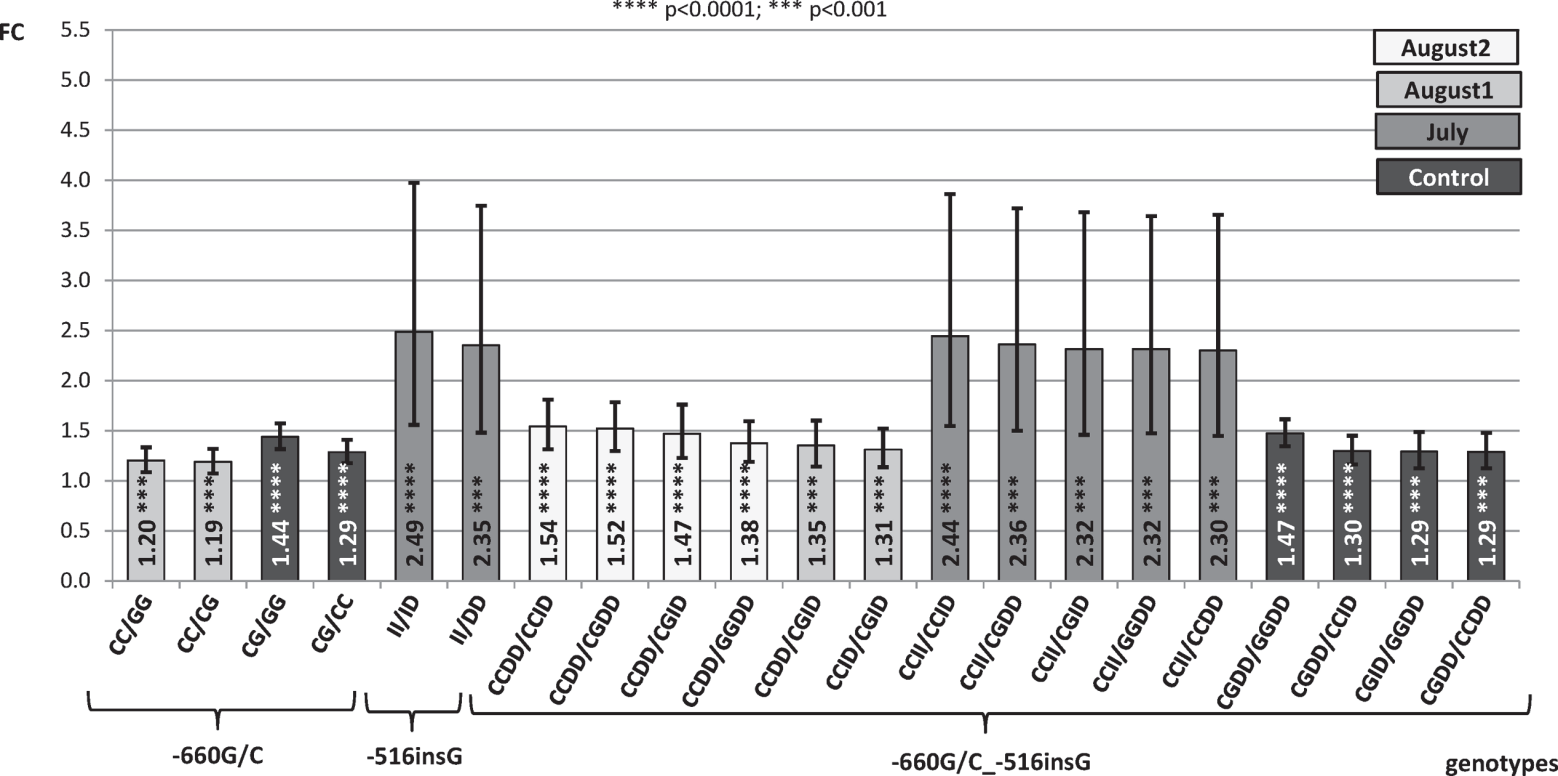
Fold change (FC) for the contrast among alternative genotypes of -660G/C, -516insG and combined genotypes -660G/C_-516insG of the *HSP90AA1* promoter within treatment normalized by *HSP90AB1*. Segments indicate the 95% confidence interval (FC_up_-FC_low_). In abscissa the FC, in ordinate genotype contrasts (I = insertion; D = deletion).

**b) Contrasts between alternative genotypes of the -516insG.** Fig. 2 shows the results for contrasts comparing guanine insertion genotypes. Only high statistically significant contrasts were showed. We could observe differences of expression between genotypes only for samples collected in July (average temperature = 26.8°C; maximum temperature = 35.0°C; minimum temperature = 16.8°C). The II_-516_ genotype showed higher expression rate than ID_-516_ (FC = 2.49, p<0.0001) and DD_-516_ (FC = 2.35 p<0.001) genotypes. However, as there were only two animals carrying the II_-516_ genotype the standard errors of the estimates were so large.

**c) Contrasts of combined genotypes -668insC_-660G/C.** Fig. 1 shows high significant contrasts among the existent combined genotypes of both polymorphisms. To facilitate the comparison with results obtained for the isolate -660G/C [16], significant contrasts for genotypes of this polymorphism were also included. In this case, for samples collected under the most extreme heat stress environmental conditions (August 2) gene expression of the combined genotypes is controlled by the genotype of the -668insC. Thus the II_-668_CC_-660_ genotype showed higher expression rates than the DD_-668_CC_-660_ (FC = 3.58) and the ID_-668_CC_-660_ (FC = 3.01). Under climatic conditions existing in August 1, lower effect of the -668insC over gene expression differences than those observed in August 2 were observed. In this case similar FC (1.6) was found in contrasts between II_-668_CC_-660_/DD_-668_CG_-660_ and II_-668_CC_-660_/DD_-668_GG_-660_. For the contrast ID_-668_CC_-660_/DD_-668_CG_-660_ and ID_-668_CC_-660_/ DD_-668_GG_-660_, FC ranged from 1.27 to 1.31. Under mild environmental temperatures (Control) the effect of the -668insC genotypes over gene expression differences was lost, and were the genotypes of the SNP -660G/C those revealing expression differences. For Control samples, animals carrying the CG_-660_ genotype showed higher expression levels than those with the GG_-660_ (FC = 1.44) and CC_-660_ (FC = 1.28 to 1.36) independently of the -668insC genotype, as it has been previously observed in [16].

**d) Contrasts of combined genotypes -660G/C_-516insG**. Fig. 2 shows high significant contrasts among the existent combined genotypes of both polymorphisms. To facilitate the comparison with results obtained for the isolate -660G/C [16], significant contrasts for genotypes of this polymorphism were also included. In significant contrasts from August 1 and 2, except in one case (CC_-660_DD_-516_/CC_-660_ID_-516_), the genotype of the -660G/C transversion seems to be the responsible of differences in the expression rate observed. Thus, animals carrying the CC_-660_ genotype independently of the -516insG one, showed higher expression levels than those with the CG_-660_ (FC from 1.31 to 1.52) and GG_-660_ (FC = 1.38). For the environmental conditions occurred when Control samples were collected, also genotypes of the -660G/C were responsible of differences observed in the expression rate of the gene (CG_-660_>GG_-660_ FC = 1.3 to 1.4 and CG_-660_>CC_-660_ FC = 1.3) as in [16].

**Figure 3:**
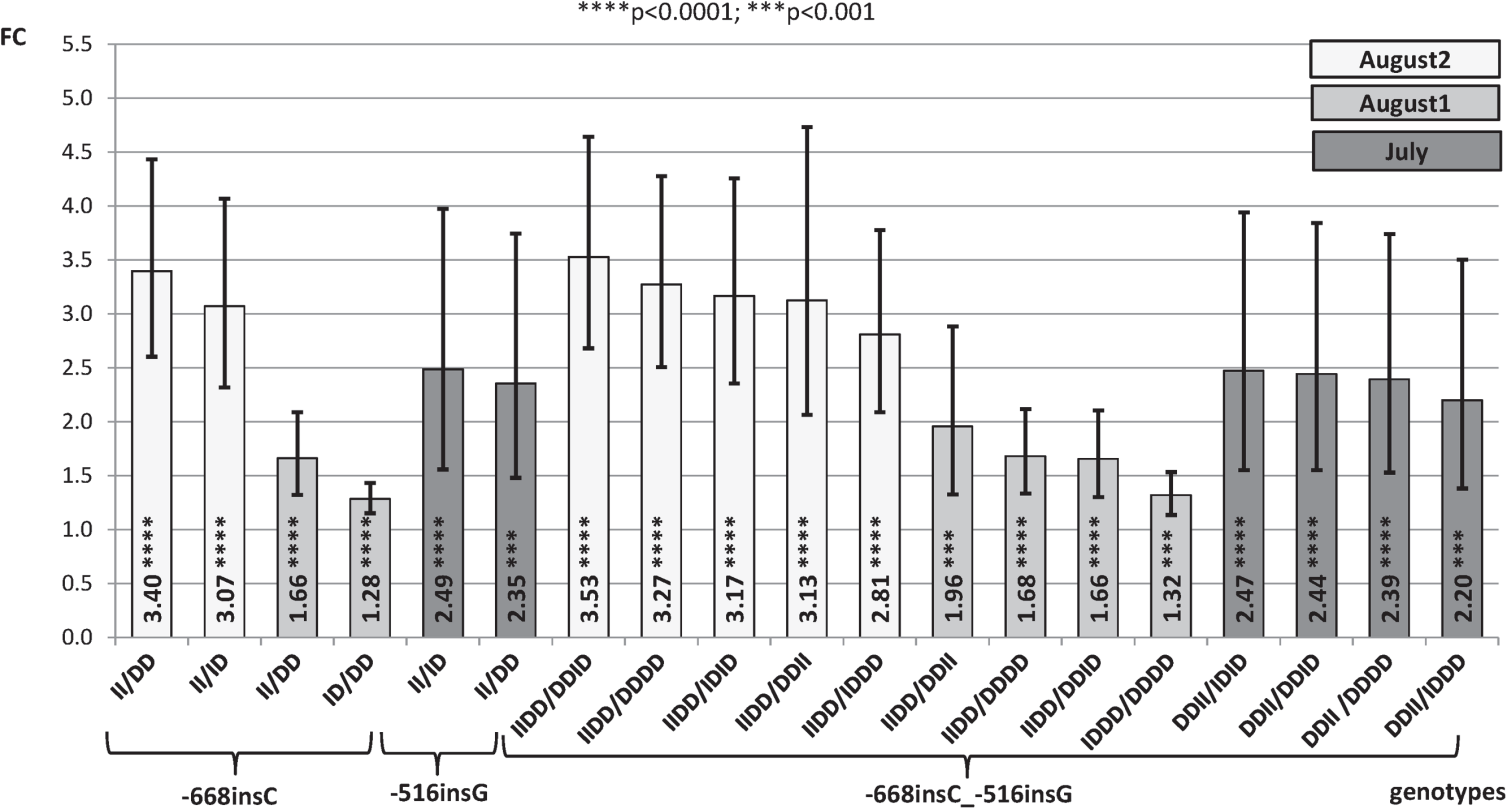
Fold change (FC) for the contrast among alternative genotypes of -668insC, -516insG and combined genotypes -668insC_-516insG of the *HSP90AA1* promoter within treatment normalized by *HSP90AB1*. Segments indicate the 95% confidence interval (FC_up_-FC_low_). In abscissa the FC, in ordinate genotype contrasts (I = insertion; D = deletion).

**e) Contrasts of combined genotypes -668insC_-516insG.** Fig. 3 shows high significant contrasts among the existent combined genotypes of both polymorphisms. In those contrasts belonging to August 1 and August 2 treatments, the preponderance of the -668insC mutation in the composed genotypes -668insC_-516insG was clear. Thus, homozygous II_-668_ genotype showed higher expression levels than the heterozygous ID_-668_ (FC = 2.81–3.17) and the homozygous DD_-668_ (1.66–3.53), independently of -516insG genotypes. Also, heterozygous ID_-668_DD_-516_ showed higher expression rate than the DD_-668_DD_-516_ (FC = 1.32). Results from July are closer to those obtained when considering the -516insG alone. It is important to emphasize that in this case many significant contrasts had high standard errors because a scarce number of animals of a particular genotype in some comparisons. These are the cases in which the II_-668_ and the II_-516_ genotypes were compared, since only five animals have the II_-668_ genotype and only two the II_-516_ one.

### Sperm DNA fragmentation as a function of environmental conditions and HSP90AA1 genotypes.

**Figure 4:**
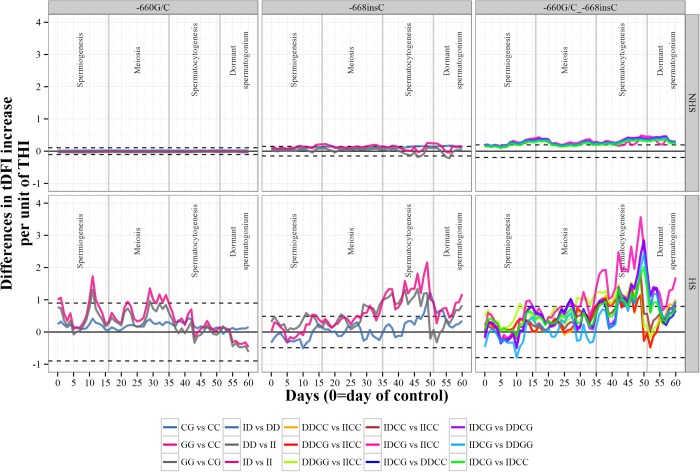
Estimates of the differences of tDFI values between genotypes of the -660G/C, -668insC and the combined genotype of both mutations depending on THI at each day of the period comprised between days 0 to 60 bsc for no heat stress (NHS) and heat stress (HS) collections. Stages of the spermatogenesis process are marked. Contrasts in the first column of the bottom legend apply to -660G/C genotypes. Contrasts in the second column of the bottom legend apply to -668insC genotypes. Contrasts in the third to fifth columns of the bottom legend apply to the combined genotypes.* *For reference values of tDFI under NHS and HS, see Table 2.

We have observed that as temperature rises, levels of sperm DNA fragmentation also increase with clear differences between non-heat stress semen collections and those conducted under heat stress conditions (Table 2). Fig. 4 shows estimates of the differences of tDFI values between genotypes of the -660G/C, -668insC and the combined genotypes of both mutations depending on THI at each day of the period comprised between days 0 to 60 bsc (before semen collection) for NHS and HS scenarios. Stages of the spermatogenesis process are marked. Bonferroni correction was applied to take into account for multiple tests.

When thermoneutral conditions occurred along the spermatogenesis process, no differences were shown among tDFI values and alternative genotypes of the -660G/C SNP. However, significant differences in tDFI values among genotypes were detected when heat stress events occur at some stages of the spermatogenesis. Thus the GG_-660_ genotype showed higher tDFI values than those observed for CC_-660_ when heat stress events occured at periods comprised between days 29 to 34 bsc (1.14 to 1.35 folds per THI unit) and 10 to 12 bsc (1.72 folds). Only for the period 10 to 12 days bsc the GG_-660_ genotype showed higher tDFI values than the CG_-660_ one (1.31 folds). No difference in tDFI values were detected between CC_-660_ and CG_-660_ genotypes.

Under NHS conditions, the ID_-668_ genotype showed greater tDFI values than the II_-668_ at some periods along the spermatogenesis process (0.22 folds for days 15 to 17; 0.19 folds for days 32 to 33; and 0.25 folds for days 49 to 52) but the magnitude of such difference was small. There were two peaks around days 46 and 56 bsc in which the II_-668_ genotype showed higher tDFI values than the DD_-668_ but even with lower magnitude than in the previous case (0.18 to 0.21 folds). When HS conditions took place, more convincing results were obtained. In this way, the ID_-668_ and DD_-668_ genotypes showed very high tDFI values than the II_-668_ (more noticeable for the ID_-668_ genotype than for the DD_-668_ one) in the period comprised between days 37–49 bsc and 29–32 bsc. Thus, for the period 37–49 days bsc ID_-668_ and DD_-668_ genotypes showed tDFI values 1.33 to 2.15 folds and 1.20 to 1.31 folds higher than the II_-668_, respectively. For the period comprised between days 29 to 32 bsc differences among genotypes were smaller than those previously described (0.82 to 0.85 folds for ID_-668_ and DD_-668_ genotypes comparing with II_-668_). These differences decreased along the spermatogenesis process disappearing around day 27 bsc.

When the combined genotypes for the polymorphisms -668insC and -660G/C were considered a mixed pattern but mainly controlled by the INDEL mutation was found. Under NHS conditions the genotype ID_-668_-CG_-660_ was the worst in terms of tDFI values compared with the remaining genotypes in the periods comprised between days 48 to 52 bsc (0.30 to 0.48 folds), 32 to 33 bsc (0.24 to 0.37 folds) and 14 to 17 bsc (0.27 to 0.43 folds). These differences had lower magnitude (0.13 to 0.27 folds) in the central stages of the spermatogenesis process (days 21 to 30). For the HS case (only significant contrasts are shown) much higher magnitude of differences among genotypes were found for the initial period of the spermatogenesis which comprises the period between 36 to 49 days bsc. Again the ID_-668_-CG_-660_ genotype was the one with the highest tDFI values (2.47 to 3.56 folds) when comparing with II_-668_-CC_-660_, DD_-668_-CC_-660_, DD_-668_-GG_-660_ and ID_-668_-CC_-660_ genotypes. Differences among genotypes were decreasing from day 36 bsc to the end of the spermiogenesis stage (1.24 to 1.92 folds). The genotype showing the lowest tDFI values was II_-668_-CC_-660_.

## Discussion

The heat shock response is one of the main prosurvival activities of cells. In particular, the sensitivity of mammalian germ cells to environmental heat stress has been extensively studied [28, 29]. Among others, cellular consequences of this stressor are protein miss-folding, DNA damaging, inhibition of DNA repair systems [30], and the inhibition of multiple processes associated with DNA replication [31–33] and the maturation of chromatin [34, 35]. To cope with these effects, cells increase the expression of heat shock proteins (HSPs). This confers a transient protection, leading to a state that is known as thermotolerance, whereby cells become more resistant to various toxic insults, including otherwise lethal temperature elevations. Moreover, HSPs are expressed, though at lower levels, under normal conditions. This observation can be explained by the fact that HSPs are molecular chaperones for protein folding that play a central role in protein homeostasis [36, 37]. There is a constant need for chaperone assistance during *de novo* protein folding and refolding of non native polypeptide chains, as the stability of cellular proteins is low and aggregation competes with productive folding even at physiological temperatures [38, 39].

Gene expression pattern of the ovine *HSP90AA1* gene has been previously studied [16, 20]. In these works, alternative genotypes of the SNP -660G/C showed differences in gene expression profile under both heat stress and thermo neutral conditions. Thus, animals carrying the CC_-660_ genotype showed higher transcription rates than those with the CG_-660_ (FC = 1.19) and the GG_-660_ (FC = 1.20) ones under heat stress conditions.

In the present study, three INDELs located at the *HSP90AA1* promoter region have been also analyzed regarding their effect over the gene expression rate. Genotypes of the -516insG only showed significant effects over the expression rate of the gene in samples collected in July. These results might involve this mutation with differences in expression under mild heat stress. However, due to the low number of animals carrying alternative genotypes of -516insG this result cannot be confirmed.

The -668insC is very close to the -660G/C transversion, which makes it a good candidate to be implied in the transcriptional regulation of the gene by itself or in a cooperative way with this SNP. The INDEL -668insC is a cytosine insertion located within a region of at least 6 cytosines which made very difficult its exact mapping and genotyping. Alternative genotypes of the -668insC have been here directly associated with differences in the transcription rate of the gene under heat stress environmental conditions but not in thermoneutral ones. Thus, animals carrying the genotype II_-668_ showed higher transcription rates than those with ID_-668_ (FC = 3.07) and DD_-668_ (FC = 3.40) genotypes for samples collected in August 2. However, much lower differences among these genotypes (II_-668_-DD_-668_ FC = 1.66 and ID_-668_-DD_-668_ FC = 1.28) for samples collected in August 1 and no differences for samples collected in July were observed. Despite maximum and average temperatures of these three collection dates are quite similar, some differences should be the clue for the results obtained in each of them. In particular, the minimum temperature in August 2 (22.2°C) was quite higher than in August 1 (16.6°C) and July (16.8°C). The minimum temperature affects one important variable related with the heat stress response, the daily thermal width (TW) which is the difference between the maximum and minimum temperatures occurring along the day. TW values were 18.2°C, 17.8°C and 11.6°C in July, August 1 and August 2 collection dates, respectively. Therefore, it seems that the heat stress response, in terms of over expression of genes involved in this metabolic pathway, rather depends on the daily temperature pattern than on the magnitude of the maximum temperature reached. That is how long is the period of time in which the environmental temperature exceeds a thermoneutral threshold.

To confirm this hypothesis, we examined the climatic conditions in a 3-day period, from 2 days before to the day of sample collection. We observed clearer differences between July samples collection with those carried out in August. For the latter, higher temperature and THI values were observed. Therefore, we confirmed that the magnitude and duration of the stress response is proportional to the dose or severity of the perturbation [40]. These facts would explain the differences in gene expression between genotypes found in the samples collected in July, August 1 and August 2.

The single effects of these two mutations (-660G/C and -668insC) increased when both polymorphisms were considered together (Fig. 1), supporting the hypothesis of a combined action in modulating the gene transcription changes. This was confirmed by a better fit of the model including both polymorphisms (S2 Table), showing that the combination of these two mutations would be able characterize better the response of the animal to thermal stress. The combined II_-668_-CC_-660_ genotype showed the highest expression levels in comparison with the remaining existent genotypes (FC from 1.27 to 3.58) under heat stress conditions. It is important to remark that in the sheep breed here studied (Manchega) these two polymorphisms showed a LD of 25%. The I_-668_-C_-660_ haplotype has a frequency of 0.14 while the D_-668_-C_-660_ and the D_-668_-G_-660_ have frequencies of 0.37 and 0.49, respectively. The haplotype I_-668_-G_-660_ does not exist in the ovine species (836 animals from 31 different sheep breeds from different locations of Europe, Africa and Asia have been genotyped, data not shown). Therefore, we cannot completely distinguish the effect of each polymorphism by itself. However, due to the proximity of both mutations, the most likely explanation to the transcriptional upregulation under heat stress is their synergistic effect.

A putative binding site for the Sp1 (specificity protein 1) transcription factor has been predicted for the sequence constituted by these two mutations (S3 Table). Sp1 is a zinc finger transcription factor that binds to GC-rich motifs of many promoters and is involved in many cellular processes, including cell differentiation, cell growth, apoptosis, immune responses, response to DNA damage, and chromatin remodeling. The highest binding affinity of Sp1 was found for the sequence containing the I_-668_ allele and the C_-660_ allele. This affinity decreases for the D_-668_–C_-660_ haplotype and disappears for the D_-668_–G_-660_ combination. The high binding affinity of Sp1 for the haplotype I_-668_–C_-660_ could be the explanation of the higher expression rate observed for the homozygous II_-668_–CC_-660_ genotype under heat stress conditions than for the remaining ones.

Whatever the mechanism of the gene transcription regulation is, it seems to be clear that genotype x environment dependent transcription rate of the *HSP90AA1* gene observed affects ram’s sperm DNA fragmentation in such a way that both events can be linked. Differences in ram’s sperm DNA fragmentation have been found by Ramón and colleagues [15] for alternative genotypes of the -660G/C transversion. In the cited work, authors observed that in animals carrying the GG genotype of this SNP sperm DNA fragmentation increases 1.3% per °C (or THI unit) when maximum environmental temperature exceeds 30°C (or average THI > 22) at days 29 to 35 and 7 to 14 before sperm collection (bsc). The same days have been reported by other studies as periods of sperm high sensitivity to heat stress [10, 41]. These periods of time coincide with the meiosis and protamination of sperm cells, respectively, which are critical thermo-sensible stages of the spermatogenesis process. Thus, CG_-660_ and GG_-660_ animals showed significant changes in their sperm DNA fragmentation values depending on environmental variables. However, based in the gene expression rates observed in the present study for the combined genotype -668insC _ -660G/C, and in the fact that both polymorphisms show a moderate LD (25%), the effect of the -660G/C SNP observed by [15] could be modulated by the presence of the -668insC.

In the present work more concluding results, regarding the linkage between gene expression and sperm DNA fragmentation, have been found when analyzing separately tDFI values of sperm samples which have been subjected or not to heat stress along the spermatogenesis process and the combined genotypes of both polymorphisms. When thermoneutral conditions surround the spermatogenesis process (sperm collected in March and May), differences of alternative genotypes of the -668insC and -660G/C mutations analyzed separately were not enough to produce significant differences in sperm DNA fragmentation and only very light when the combined genotypes -668insC _ -660G/C (Fig. 4) were considered (less than 0.5 tDFI units). However when heat stress conditions were present along or at some stages of the spermatogenesis process (sperm collected in June, July, August and October) different results were observed when considering the -668insC and -660G/C genotypes individually or combined, not only in the magnitude of the differences in tDFI values observed among genotypes but also in the spermatogenesis stage where heat stress has greater effect over sperm DNA fragmentation measured after 48h of 37°C heating after ejaculate collection.

Our results confirmed that both polymorphisms are involved in the effect that climatic conditions has over sperm DNA fragmentation and that previous results [15] associated to the -660G/C were masking the effect of the INDEL -668insC, which resulted to be more important. Fig. 4 shows similar peaks at the spermiogenesis and meiosis stages to those observed for the -660G/C alone. However, the critical stage in terms of heat stress effect over sperm DNA fragmentation differences moved to the spermatocytogenesis stage in where the maximum tDFI differences between genotypes were observed. These differences ranged from 1.27 to 1.32 folds for the DD_-668_GG_-660_ vs. II_-668_CC_-660_, and from 1.92 to 3.56 folds for the ID_-668_CG_-660_ vs. II_-668_CC_-660_. Differences in the gene expression rate for these same genotypes were also high (FC = 1.6 to 3.1) when heat stress conditions (August 2 and August 1) were present. Therefore, it seems reasonable to consider that both events are correlated, but in what way?

It is well known that there are differences in the genes transcription rate in the stages (cell types) of the spermatogenesis process [41–43] and also in the heat stress sensitivity of the different cell types involved [10, 41, 44]. In the spermatocytogenesis stage (mitosis), spermatogonia and primary spermatocytes have high transcription levels as occurs in other undifferentiated cells. However these levels decay in meiotic cells (secondary spermatocytes and spermatids) and in mature spermatozoa. Moreover, cell-specific genes are transcribed at each stage of the spermatogenesis process [42]. In rats, the expression levels of the *HSP90AA1* gene decrease drastically (80%) during the early phases of the spermatogenesis reaching undetectable levels in the more mature germ cells [42] as it has been observed for our group in sheep spermatozoa (data not shown). During germ cell development, different spermatogenic cell types showed remarkable variation in their susceptibility to heat stress being spermatogonia and spermatozoa the most thermotolerant cells while pachytene spermatocytes and early spermatids are more susceptible to heat [3, 7].

Taking into account this background, we can make some hypothesis around the results here obtained regarding the expression levels observed for the *HSP90AA1* gene in animals carrying alternative combined genotypes of the -668insC_ -660G/C mutations and the variation of the tDFI values observed in the spermatozoa of these same animals depending on heat stress events occurring along the spermatogenesis process. The highest differences in tDFI values among -668insC_ -660G/C combined genotypes were observed when the THI threshold was exceeded during the spermatocytogenesis stage, independently from heat stress events occurring in posterior phases of the spermatogenesis process. Heat stress at this stage induces the expression of the *HSP90AA1* gene. Thus unfavorable genotypes in terms of gene expression induction (ID_-668_GC_-660_, DD_-668_GG_-660_) do not produce enough mRNA (mRNAs are stored as messenger ribonucleoprotein particles [45]) and Hsp90α protein to cope with future thermal stress which might occur in posterior stages in which transcriptional activity is reduced and cell types and molecular processes are more sensible to heat (spermatocytes in pachytene and spermatids protamination).When THI threshold was exceeded in the meiosis and spermiogenesis stages differences in tDFI values of alternative combined genotypes of -668insC_ -660G/C are much lower than those observed in the previous case described maybe due to the limited transcriptional activity of the cellular types here involved. Two peaks of higher differences corresponding to meiosis and protamination could indicate the importance of past (selective translation of stored mRNAs [46]) and present (limited) expression rates of the *HSP90AA1* gene to protect the meiotic process and produce an optimal exchange of histones by protamines [46, 47] to achieve an optimal spermatozoa DNA packaging.

Therefore, optimal expression rates of favorable genotypes of the *HSP90AA1* gene induced by heat stress events seem to be related with a higher ability of mature spermatozoa to cope with the effects that high temperatures exert over their DNA fragmentation when they are subjected to 37°C for 48h. This ability must consist essentially in a better packaging of the sperm DNA (efficient protamination) during the spermiogenesis process which would be favored by higher amounts of Hsp90α, translated at this moment or stored in the past. In bulls, the DNA fragmentation index (DFI), has been positively correlated with the percentage of spermatozoa that showed low protamine content [Fortes et al, 2014]. However, other roles of the Hsp90α related with the cellular defense against other sources of stress (i.e. oxidative stress) and proteostasis maintenance [48] must not be discarded to preserve spermatozoa DNA from injuries.

Results here obtained lead us to question if heat stress events occurring at initial stages of the spermatogenesis process would be selectively advantageous to protect cell types of subsequent stages, which have worse heat stress response in terms of transcription ability, from injuries caused by heat or other sources of stress. Relative to this idea, it is important to remark that sheep is a short day breeder whose favorable reproductive period begins when the days shorten (fewer hours of light). This period comes after the hottest months, and so, we could expect that those animals with a favorable genotype in terms of heat resistance were more fertile.

Future functional *in vitro* studies, can contribute to elucidate which polymorphism(s) and transcription factor(s) are involved in the expression differences observed in this gene as response to environmental conditions. Also the methylation pattern of the *HSP90AA1* promoter would provide information about other possible mechanism for the regulation of the gene expression. Finally, association studies among sperm DNA fragmentation and *HSP90AA1* genotypes with ram’s fertility will contribute to determine the involvement of this gene in ram’s reproductive cells thermo sensibility and its consequences over their reproductive efficiency.

## Supporting Information

S1 Fig *HSP90AA1* promoter region containing the polymorphism positions. In dark grey the heat shock element (HSE) binding heat shock factor 1 (HSF1). In light grey the TATA box sequence.
Polymorphisms identifications are the following: ^1^-704insAA; ^2^-668insC (rs397514115.2); ^3^-667insC (rs397514115.1); ^4^-660G/C (rs397514116); ^5^-601A/C (rs397514117); ^6^-528A/G (rs397514269); ^7^-524G/T (rs397514270); ^8^-522A/G (rs397514271); ^9^-516insG (rs397514268); ^10^-468G/T (rs397514272); ^11^-444A/G (rs397514273).
(DOCX)

S1 Table List of primers used to amplify and/or sequence the *HSP90AA1* gene polymorphisms. (DOC)

S2 Table Goodness of fit criteria for models used to analyze expression data. (DOC)

S3 Table Putative transcription factors predicted for the -668insC and -660G/C polymorphisms by Chip Mapper [24]. (DOCX)

## References

[1] Verghese J, Abrams J, Wang Y, Morano KA (2012) Biology of the heat shock response and protein chaperones: budding yeast (Saccharomyces cerevisiae) as a model system. Microbiol Mol Biol Rev 76: 115–158. 10.1128/MMBR.05018-11 22688810 PMC3372250

[2] Sreedhar AS, Kalmar E, Csermely P, Shen YF (2004) Hsp90 isoforms: functions, expression and clinical importance. FEBS Lett 562: 11–15. 10.1016/S0014-5793(04)00229-7 15069952

[3] Grad I, Cederroth CR, Walicki J, Grey C, Barluenga S, et al (2010) The molecular chaperone Hsp90alpha is required for meiotic progression of spermatocytes beyond pachytene in the mouse. PLoS One 5: e15770 10.1371/journal.pone.0015770 21209834 PMC3013136

[4] De la Fuente M, Valera S, Martinez-Guitarte JL (2012) ncRNAs and thermoregulation: a view in prokaryotes and eukaryotes. FEBS Lett 586: 4061–4069. 10.1016/j.febslet.2012.10.018 23098758

[5] Ulberg LC (1958) The influence of high temperature on reproduction. J Hered 49.

[6] Hales BF, Aguilar-Mahecha A, Robaire B (2005) The stress response in gametes and embryos after paternal chemical exposures. Toxicol Appl Pharmacol 207: 514–520. 10.1016/j.taap.2004.12.021 15982682

[7] Setchell BP (2006) The effects of heat on the testes of mammals. Anim Reprod 3: 81–91.

[8] Paul C, Melton DW, Saunders PT (2008) Do heat stress and deficits in DNA repair pathways have a negative impact on male fertility? Mol Hum Reprod 14: 1–8. 10.1093/molehr/gam089 18175790

[9] Banks S, King SA, Irvine DS, Saunders PTK (2005) Impact of a mild scrotal heat stress on DNA integrity in murine spermatozoa. Reproduction 129: 505–514. 10.1530/rep.1.00531 15798026

[10] Pérez-Crespo M, Pintado B, Gutierrez-Adán A (2008) Scrotal heat stress effects on sperm viability, sperm DNA integrity and the Offspring sex ratio in mice. Molecular Reproduction and Development 75: 40–47. 10.1002/mrd.20759 17474098

[11] Fleming JS, Yu F, McDonald RM, Meyers SA, Montgomery GW, Smith JF, Nicholson HD (2004) Effects of scrotal heating on sperm surface protein PH-20 expression in sheep. Mol Reprod Dev 68: 103–114. 10.1002/mrd.20049 15039954

[12] Sailer BL Sarkar LJ, Bjordahl JA, Jost LK, Evenson DP (1997) Effects of heat stress on mouse testicular cells and sperm chromatin structure. J Androl 18: 294–301. 9203058

[13] Ward WS (2010) Function of sperm chromatin structural elements in fertilization and development. Mol Hum Reprod 16: 30–36. 10.1093/molehr/gap080 19748904 PMC2790366

[14] Dominguez K, Arca CR, Ward WS (2011) The Relationship Between Chromatin Structure and DNA Damage in Mammalian Spermatozoa. In: Zini A, Agarwal A, editors. Sperm Chromatin: Springer New York pp. 61–68. 10.1007/978-1-4419-6857-9_4

[15] Ramón M, Salces-Ortiz J, González C, Pérez-Guzmán MD, Garde J, et al (2014) Influence of the Temperature and the Genotype of the HSP90AA1 Gene over Sperm Chromatin Stability in Manchega Rams. Plos One 9 e86107 10.1371/journal.pone.0086107 24465903 PMC3897619

[16] Salces-Ortiz J, González C, Moreno-Sanchez N, Calvo JH, Perez-Guzman MD, et al (2013) Ovine HSP90AA1 expression rate is affected by several SNPs at the promoter under both basal and heat stress conditions. Plos One 8 e66641 10.1371/journal.pone.0066641 23826107 PMC3691178

[17] Marcos-Carcavilla A, Calvo JH, González C, Moazami-Goudarzi K, Laurent P, et al (2008) Structural and functional analysis of the HSP90AA1 gene: distribution of polymorphisms among sheep with different responses to scrapie. Cell Stress & Chaperones 13: 19–29. 10.1007/s12192-007-0004-2 18347938 PMC2666211

[18] Marcos-Carcavilla A, Moreno C, Serrano M, Laurent P, Cribiu EP, A et al (2010) Polymorphisms in the HSP90AA1 5’ flanking region are associated with scrapie incubation period in sheep. Cell Stress & Chaperones 15: 343–349. 10.1007/s12192-009-0149-2 19838832 PMC3082647

[19] Oner Y, Calvo JH, Elmaci C (2013) Investigation of the genetic diversity among native Turkish sheep breeds using mtDNA polymorphisms. Trop Anim Health Prod 45: 947–951. 10.1007/s11250-012-0313-z 23135986

[20] Marcos-Carcavilla A, Mutikainen M, González C, Calvo JH, Kantanen J, et al (2010) A SNP in the HSP90AA1 gene 5’ flanking region is associated with the adaptation to differential thermal conditions in the ovine species. Cell Stress & Chaperones 15: 67–81. 10.1007/s12192-009-0123-z 19496025 PMC2866970

[21] Marai IFM, El-Darawany AA, Fadiel A, Abdel-Hafez MAM (2007) Physiological traits as affected by heat stress in sheep—a review. Small Rum Res 71:1–12. 10.1016/j.smallrumres.2006.10.003

[22] Purcell S, Neale B, Todd-Brown K, Thomas L, Ferreira MAR, et al (2007) PLINK: A tool set for whole-genome association and population-based linkage analyses. American Journal of Human Genetics 81: 559–575. 10.1086/519795 17701901 PMC1950838

[23] Hill WG, Robertson A (1968) Linkage disequilibrium in finite populations. Theoretical and Applied Genetics 38:226–231. 10.1007/BF01245622 24442307

[24] Marinescu VD, Kohane IS, Riva A (2005) The MAPPER database: a multi-genome catalog of putative transcription factor binding sites. Nucleic Acids Res 33: D91–97. 10.1093/nar/gki103 15608292 PMC540057

[25] Steibel JP, Poletto R, Coussens PM, Rosa GJ (2009) A powerful and flexible linear mixed model framework for the analysis of relative quantification RT-PCR data. Genomics 94: 146–152. 10.1016/j.ygeno.2009.04.008 19422910

[26] Evenson DP, Jost LK, Marshall D, Zinaman MJ, Clegg E, et al (1999) Utility of the sperm chromatin structure assay (SCSA) as a diagnostic and prognostic tool in the human fertility clinic. Hum Reprod 14: 1039–1049. 10.1093/humrep/14.4.1039 10221239

[27] Team RC (2013) A language and environmental for statistical computing. In: Computing RFfS, editor. Vienna, Austria.

[28] Kim B, Park K, Rhee K (2013) Heat stress response of male germ cells. Cell Mol Life Sci 70: 2623–2636. 10.1007/s00018-012-1165-4 23007846 PMC11113252

[29] Hansen PJ (2009) Effects of heat stress on mammalian reproduction. Philos Trans R Soc Lond B Biol Sci 364: 3341–3350. 10.1098/rstb.2009.0131 19833646 PMC2781849

[30] Stecklein SR, Kumaraswamy E, Behbod F, Wang W, Chaguturu V, et al (2012) BRCA1 and HSP90 cooperate in homologous and non-homologous DNA double-strand-break repair and G2/M checkpoint activation. Proc Natl Acad Sci U S A 109: 13650–13655. 10.1073/pnas.1203326109 22869732 PMC3427093

[31] De Carcer G (2004) Heat shock protein 90 regulates the metaphase-anaphase transition in a polo-like kinase-dependent manner. Cancer Res 64: 5106–5112. 10.1158/0008-5472.CAN-03-2214 15289312

[32] McClellan AJ, Xia Y, Deutschbauer AM, Davis RW, Gerstein M, et al (2007) Diverse cellular functions of the Hsp90 molecular chaperone uncovered using systems approaches. Cell 131: 121–135. 10.1016/j.cell.2007.07.036 17923092

[33] Velichko AK, Markova EN, Petrova NV, Razin SV, Kantidze OL (2013) Mechanisms of heat shock response in mammals. Cell Mol Life Sci 70: 4229–4241. 10.1007/s00018-013-1348-7 23633190 PMC11113869

[34] Zhao R, Davey M, Hsu YC, Kaplanek P, Tong A, et al (2005) Navigating the chaperone network: an integrative map of physical and genetic interactions mediated by the hsp90 chaperone. Cell 120: 715–727. 10.1016/j.cell.2004.12.024 15766533

[35] Ruden DM, Lu X (2008) Hsp90 affecting chromatin remodeling might explain transgenerational epigenetic inheritance in Drosophila. Curr Genomics 9: 500–508. 10.2174/138920208786241207 19506739 PMC2691676

[36] Freeman BC, Michels A, Song J, Kampinga HH, Morimoto RI (2000) Analysis of molecular chaperone activities using in vitro and in vivo approaches. Methods Mol Biol 99:393–419. 10.1385/1-59259-054-3:393 10909095

[37] Diller KR (2006) Stress protein expression kinetics. Annu Rev Biomed Eng 8: 403–424. 10.1146/annurev.bioeng.7.060804.100449 16834562

[38] Kerner MJ, Naylor DJ, Ishihama Y, Maier T, Chang HC, et al (2005) Proteome-wide analysis of chaperonin-dependent protein folding in Escherichia coli. Cell 122:209–220. 10.1016/j.cell.2005.05.028 16051146

[39] Mayer MP (2010) Gymnastics of molecular chaperones. Mol Cell 39: 321–331. 10.1016/j.molcel.2010.07.012 20705236

[40] Gasch AP, Spellman PT, Kao CM, Carmel-Harel O, Eisen MB, et al (2000) Genomic expression programs in the response of yeast cells to environmental changes. Mol Biol Cell 11: 4241–4257. 10.1091/mbc.11.12.4241 11102521 PMC15070

[41] Paul C, Murray AA, Spears N, Saunders PTK (2008) A single, mild, transient scrotal heat stress causes DNA damage, subfertility and impairs formation of blastocysts in mice. Reproduction 136: 73–84. 10.1530/REP-08-0036 18390691

[42] Aguilar-Mahecha A, Hales BF, Robaire B (2001) Expression of stress response genes in germ cells during spermatogenesis. Biol Reprod 65: 119–127. 10.1095/biolreprod65.1.119 11420231

[43] Vibranovski MD, Chalopin DS, Lopes HF, Long M, Karr TL (2010) Direct evidence for postmeiotic transcription during Drosophila melanogaster spermatogenesis. Genetics 186: 431–433. 10.1534/genetics.110.118919 20610406 PMC2940308

[44] Rockett JC, Mapp FL, Garges JB, Luft JC, Mori C, et al (2001) Effects of hyperthermia on spermatogenesis, apoptosis, gene expression, and fertility in adult male mice. Biol Reprod 65: 229–239. 10.1095/biolreprod65.1.229 11420244

[45] Hecht NB (1998) Molecular mechanisms of male germ cell differentiation. Bioessays 20: 555–561. 10.1002/(SICI)1521-1878(199807)20:7<555::AID-BIES6>3.0.CO;2-J 9723004

[46] Rathke C, Baarends WM, Awe S, Renkawitz-Pohl R (2014) Chromatin dynamics during spermiogenesis. Biochim Biophys Acta 1839: 155–168. 10.1016/j.bbagrm.2013.08.004 24091090

[47] Campos EI, Fillingham J, Li G, Zheng H, Voigt P, et al (2010) The program for processing newly synthesized histones H3.1 and H4. Nat Struct Mol Biol 17: 1343–1351. 10.1038/nsmb.1911 20953179 PMC2988979

[48] Erlejman AG, Lagadari M, Toneatto J, Piwien-Pilipuk G, Galigniana MD (2014) Regulatory role of the 90-kDa-heat-shock protein (Hsp90) and associated factors on gene expression. Biochim Biophys Acta 1839: 71–87. 10.1016/j.bbagrm.2013.12.006 24389346

