# Supplementary material for: Differences in the Ovine HSP90AA1 Gene Expression Rates Caused by Two Linked Polymorphisms at Its Promoter Affect Rams Sperm DNA Fragmentation under Environmental Heat Stress Conditions
Source: PLoS One. 2015 Feb 11;10(2):e0116360. doi: 10.1371/journal.pone.0116360 (PMC4324765; doi:10.1371/journal.pone.0116360)
Supplement: S1 Fig — In dark grey the heat shock element (HSE) binding heat shock factor 1 (HSF1). In light grey the TATA box sequence. Polymorphisms identifications are the following: 1-704insAA; 2-668insC (rs397514115.2); 3-667insC (rs397514115.1); 4-660G/C (rs397514116); 5-601A/C (rs397514117); 6-528A/G (rs397514269); 7-524G/T (rs397514270); 8-522A/G (rs397514271); 9-516insG (rs397514268); 10-468G/T (rs397514272); 11-444A/G (rs397514273). (DOCX) [file pone.0116360.s001.docx]

**Supplemental Figure 1.** *HSP90AA1* promoter region containing the polymorphism positions. In dark grey the heat shock element (HSE) binding heat shock factor 1 (HSF1). In light grey the TATAbox sequence.

Polymorphisms identifications are the following: ^1^-704insAA; ^2^-668insC (rs397514115.2); ^3^-667insC (rs397514115.1); ^4^-660G/C (rs397514116); ^5^-601A/C (rs397514117); ^6^-528A/G (rs397514269); ^7^-524G/T (rs397514270); ^8^-522A/G (rs397514271); ^9^-516insG (rs397514268); ^10^-468G/T (rs397514272); ^11^-444A/G (rs397514273).

**>gi|115503918|gb|DQ983231.1| Ovis aries heat shock protein alpha (HSPCA) gene**

CCTGGCGGGCTACCGTCGCAGAGTTGGACACGACTGAGCGACTAAGCACAGCACAAAGTAGGTACAAAGGCTCAAAGAACTAAGCAAAGGC

CGGGAGAATTCGGGACTGTGTCCCCTTTTAACATTCCCCGCGCCACCGCTCCCCCTGCCTTGGGCGCAATCGGGCCTAAGGACAGGACGAG

TCCCCATACTGGAGTGGCGGGGGAATGGTGGTTTGTTCCAGGGCCCACCTCTGGTTCAGAACCAGCCGAATAGACCCACGAGGCTCTGGAG

TTCCCCTCTGCTCCCCCTGCACACACGCTGGTCACCCCTCGCTCACAGACCGCTGCACCCCACAACCAGGCTGGGGCTCTGAGCCTCCGCG

TTAACGAGGCTCTGGCAGGCACTTGTTGCCCGAATTCCTAGGGCCCTGAACCTATAACCGCTGCCCCTGGCA(**AA/--)^1^**GAGATGCGCCCT

AGACCCTCTAATCGCTGCCCGGA**(-/C)^2^(-/C)^3^**CCCCCC**(G/C)^4^**AAACCCCTAACCATGGACCTGAGCCTGCGATCAATGCCCCAAGCAT

ATACCTGTGACA**(A/C)^5^**GGGACTGGACCCCTCACCCCGGCTTTGATCCCCTAAGCGCGGCCTCAAGGCTCGAAACCTAGCCCCAGGCCC**(A/G)^6^**

TAG**(G/T)^7^**C**(A/G)^8^**GGGGG**(-/G)^9^**TCCCGATGCTGGGGGGATCCGGAGCGCACACTATCTGGGGACACGCTT**(G/T)^10^**AACCACCGAGAG

TTTACTTTTCT**(A/G)^11^**GACTTCCCAGAGCTGCTGGCACGGGCGATCAGCCCCCGGGGCCTGGACAGAGGTTTGGGTCGGGGGAGCGTTAGG

CCGACGCGCGCCAAGCGGGGTTGGCGGGCCAGCGGTGTGAGGCCGGCGGGGCGCAGAACGCCGCGCGCCGGGGTAGGTAGGGCTGGGAACGG

CGGCACCGGAAGGCGCGAGACCCGCCGGAACGCTCCGGATAGCGCAGGCGCACTGCGGCGGGCGCGGGCACGCTTGGCACGCAGGCGCCCTG

AGGCGTGGGAGCGCGCGGCGCGGCCCCGAGGAGGGGGCGGGCGGCCTCCAGTGCGCAGGCGCAGGCGCGCGGCCGCTCCGGCGGTT**GGGGAGG**

**GTTCTTCCGGAAGGTTCGGGAGGCTTCTGGAA**AAGGCGCCGCGCGCTGGGCGGGCCCGCCTC**TATATAAG**GGCGGCGCGGGGGCGGCGCGC

CAGTTGCCTCTGCGTCCCGGTGCTGCGTCGCTGCGGAGCCCGTGCTAGTCCTTCAGCCAAGGTGAGGGGAGGCGGTTGTAGGGGGACGCGGG

GATCCACGGGCGGGTGGGCGGCGGCGAGGGCAGCCCCCGGAGCCCCCTGAGGCCGGCGGGCCCGGGGCGGCCGTGATTAACCCGGTAGGCCT

CGGGCCTGCGGCGGTCTCCCGTCCCCTCCCCCCCGCCCTCCCCCCGCCGTCGGGAGCGAGTGGGCCCCGGGGCTCCGGGGTCGTGCGGGATG

GTGAGGGCCGGGGTCTGCGGCCCCGGAGGGGGGTCCCCGGGCCGCGGGTGGGGGCGCCCGGGCCGAGCGGCGGCCGTGCGGCCGGCGTGGGC

GGCGGCGGCAGCGGCGGCGCCCGGCCCCCGGAATGAGGTCATCCTTTGTCAGCCGGCGCCTCCCGCGCGCCCCGGTCTCGGGAGCGGGAGCG

AGACGCGGCGGGGAGGGGTCCGGCCGGTGGCCTCACGGGCTCCGCGTTCCCCGGCAGATGCCCGAGGAGACCCAGGCCCAGGACCAGCCGAT

GGAGGAGGAGGAGGTGGAGACGTTCGCCTTCCAGGCGGAAATTGCCCAGTTGATGTCACTGATCATCAACACTTTCTACTCGAATAAAGAGA

TTTTCCTGAGGGAGCTGATTTCAAACTCGTCAGACGT
