# Supplementary material for: Differences in the Ovine HSP90AA1 Gene Expression Rates Caused by Two Linked Polymorphisms at Its Promoter Affect Rams Sperm DNA Fragmentation under Environmental Heat Stress Conditions
Source: PLoS One. 2015 Feb 11;10(2):e0116360. doi: 10.1371/journal.pone.0116360 (PMC4324765; doi:10.1371/journal.pone.0116360)
Supplement: S1 Table — (DOC) [file pone.0116360.s002.doc]

**Supplemental Table 1.** List of primers used to amplify and/or sequence the *HSP90AA1* gene polymorphisms.

|  | 5’ flanking region |  | Amplicon size |
| --- | --- | --- | --- |
| Forward (5’-3’) | CGAGGCTCTGGCAGGCACTTGTTG |  | 499pb |
| Reverse (5’-3’) | GCCGCCGTTCCCAGCCCTACCT |  |  |
| Sequencing -668insC and -667insC (5’-3’)  Sequencing -516insG (5’-3’) | GCTAGGTTTCGAGCCTTGAGG  AAGCGTGTCCCCAGATAGTG |  |  |
