# Supplementary material for: Differences in the Ovine HSP90AA1 Gene Expression Rates Caused by Two Linked Polymorphisms at Its Promoter Affect Rams Sperm DNA Fragmentation under Environmental Heat Stress Conditions
Source: PLoS One. 2015 Feb 11;10(2):e0116360. doi: 10.1371/journal.pone.0116360 (PMC4324765; doi:10.1371/journal.pone.0116360)
Supplement: S2 Table — (DOC) [file pone.0116360.s003.doc]

**Supplemental Table 2.** Goodness of fit criteria for models used to analyze expression data.

| **Fit statistics*** | **-668insC** | **-660G/C** | **-668insC_-660G/C** |
| --- | --- | --- | --- |
| **Likelihood-ratio test** | 11720 | 11809 | 11607 |
| **AIC** | 11732 | 11821 | 11619 |
| **AICC** | 11732 | 11821 | 11619 |
| **BIC** | 11749 | 11838 | 11636 |

*the smaller, the better
