# Supplementary material for: Differences in the Ovine HSP90AA1 Gene Expression Rates Caused by Two Linked Polymorphisms at Its Promoter Affect Rams Sperm DNA Fragmentation under Environmental Heat Stress Conditions
Source: PLoS One. 2015 Feb 11;10(2):e0116360. doi: 10.1371/journal.pone.0116360 (PMC4324765; doi:10.1371/journal.pone.0116360)
Supplement: S3 Table — (DOCX) [file pone.0116360.s004.docx]

**Supplemental Table 2**. Putative transcription factors predicted for the -668insC rs397514115.2 and -660G/C polymorphisms by Chip Mapper [22].

| GGA(-_-668_)CCCCCC(G_-660_)AAACCCCTAA | | | | | |
| --- | --- | --- | --- | --- | --- |
| **Factor** | **Strand** | **Start** | **End** | **Score** | **E-value** |
| LXR | - | 1 | 18 | 4.5 | 12 |
| Zic3 | - | 2 | 9 | 3.3 | 17 |
| Spz1 | - | 7 | 17 | 1.2 | 13 |
| GBF | - | 12 | 20 | 1.7 | 20 |
| GGA(-_-668_)CCCCCC(C_-660_)AAACCCCTAA | | | | | |
| **Factor** | **Strand** | **Start** | **End** | **Score** | **E-value** |
| Zic3 | - | 2 | 9 | 3.3 | 17 |
| **SP1** | **-** | **3** | **11** | **0.8** | **13** |
| Lyf-1 | - | 5 | 13 | 3 | 16 |
| LIM1 | + | 6 | 17 | 2.2 | 18 |
| RREB-1 | + | 7 | 20 | 4.3 | 15 |
| GBF | - | 12 | 20 | 1.7 | 20 |
| GGA(C_-668_)CCCCCC(C_-660_)AAACCCCTAA | | | | | |
| **Factor** | **Strand** | **Start** | **End** | **Score** | **E-value** |
| Zic3 | - | 2 | 9 | 3.3 | 17 |
| **SP1** | **-** | **3** | **11** | **1.9** | **4.3** |
| Lyf-1 | - | 6 | 14 | 3 | 16 |
| LIM1 | + | 7 | 18 | 2.2 | 18 |
| RREB-1 | + | 8 | 21 | 4.3 | 15 |
| GBF | - | 13 | 21 | 1.7 | 20 |
